# Leaf age and light stress affect the ability to diagnose P status in field grown potatoes

**DOI:** 10.3389/fpls.2023.1100318

**Published:** 2023-04-20

**Authors:** Stine Le Tougaard, Augusta Szameitat, Pauline Møs, Søren Husted

**Affiliations:** Section for Plant and Soil Science, Department of Plant and Environmental Sciences, University of Copenhagen, Copenhagen, Denmark

**Keywords:** *Solanum tuberosum*, chlorophyll *a* fluorescence, phosphorus deficiency, nutrient remobilization, critical nutrient concentration

## Abstract

Phosphorus (P) deficiency is a global issue which can severely impact the yield of crops, including the P demanding and important food crop potato. Diagnosis of P status directly in the field can be used to adapt P fertilization strategies to the needs of the evolving crop during the growing season and is often estimated by analyzing P concentrations in leaf tissue. In this study, we investigate how diagnosis of P status in field grown potato plants is affected by leaf position and time of measurement in a randomized block experiment. The concentrations of many essential plant nutrients are highly dynamic, and large differences in nutrient concentrations were found in potato leaves depending on leaf age and time of sampling. During tuber initiation, P concentrations decreased in a steep gradient from the youngest leaves (0.8%) towards the oldest leaves (0.2%). The P concentrations in the youngest fully expanded leaf decreased by 25-33% within just 7 days, due to a high remobilization of P from source to sink tissue during crop development. 40 days later P concentrations in all leaves were near or below the established critical P concentration of 0.22%. The P concentration in leaf tissue thus depends on sampling time and leaf position on the plant, which in a practical setting might prevent a meaningful interpretation in terms of fertilizer recommendation. The chlorophyll *a* fluorescence parameter “P-predict”, derived from the fluorescence transients, is an alternative to the classical chemical analysis of nutrient concentrations in leaf tissue. P-predict values serve as a proxy for the bioavailable P pool in the leaf and can be measured directly in the field using handheld technology. However, in conditions of high solar irradiation, the P-predict values of the most light-exposed leaf positions, i.e. the younger leaves, were found to be severely impacted by photoinhibition, preventing accurate characterization of the P status in potatoes. Shading the plants can reverse or prevent photoinhibition and restore the diagnostic capabilities of the P-predict approach.

## 1 Introduction

Potatoes are the third most important world crop, supplying food to more than a billion people worldwide (Birch et al., 2012). It is a nutritionally valuable staple crop used for various purposes, i.e. fresh consumption, processed food products and starch extraction, with many specific quality requirements which can be influenced by the availability of different plant nutrients. Potato is a highly phosphorus (P) demanding crop, and adequate P supply throughout the entire growth period is crucial for optimal yields and product quality. In the early growing season during the tuber initiation stage, P has a significant effect on tuber setting (Jenkins and Ali, 2000). The highest quantity of P is taken up during tuber bulking in the mid- to late growing season, and continues in the tuber maturation phase, where it improves tuber maturity (Rosen et al., 2014). P uptake continues even after uptake of other nutrients, such as nitrogen (N) and potassium (K), have ceased (Rosen et al., 2014). During tuber bulking, P has a critical role in carbon partitioning and starch synthesis, where key enzymes such as ADP-glucose pyrophosphorylase (AGPase) are tightly regulated by the phosphate concentration in amyloplasts. Moreover, P is essential for regulation of starch synthesis through phosphorylation of enzymes and carbohydrate intermediates (Geigenberger, 2011; MacNeill et al., 2017). Furthermore, phosphate is covalently bound in amylopectin of storage starch species in root and tuber crops, most prevalent in potato, where the degree of phosphate substitution affects the properties of potato starch paste (Noda et al., 2007).

Regulation of biochemical processes through phosphorylation is a central function of P in plant cells, but the most essential role of P is found in adenosine triphosphate (ATP) (de Bang et al., 2021). Hydrolysis of ATP is the primary source of biochemical energy in the plant and is necessary for generating the proton motive force (PMF) required for loading sucrose into the phloem. Phloem loading, translocation and unloading of sucrose is essential for the movement of photosynthates from leaves to tubers, and to synthesize and store starch in the tubers (Hammond and White, 2008). Phloem loading is also dependent on the essential nutrients Potassium (K) and Magnesium (Mg). During inadequate supply of either of these nutrients, carbohydrate distribution is affected, and sucrose accumulates in potato leaves (Cakmak et al., 1994; Ceylan et al., 2016; Koch et al., 2019a). This is related to the important role of Mg as an activator of ATP, making it a substrate for ATPase to generate the PMF (Hermans et al., 2005). Mg furthermore serves crucial functions in photosynthetic light harvesting as a co-factor of chlorophyll (Verbruggen and Hermans, 2013).

Both Mg and, especially, K serve as osmotic and enzymatic regulators within the plant. K establishes the osmotic pressure necessary for transport of sucrose via the phloem, and it regulates pH via charge balancing across membranes, which is essential to drive ATP production and optimize enzyme activity. In particular, K is an activator of starch synthase which is required for starch synthesis. It therefore has a central role in establishing tuber and starch yields (Koch et al., 2019b). K also influences quality traits such as storability and cooking type, which is mainly determined by starch content, and a sufficient supply helps reduce after-cooking darkening, black spot bruise and formation of acrylamide (Naumann et al., 2019). K is typically the most abundant element in potatoes at a concentration up to 6% in the leaves and 1.7-2.5% in the tubers (Koch et al., 2019b), with a maximum K uptake during tuber initiation and bulking and close to zero uptake during the maturation phase (Horneck and Rosen, 2008).

In addition to the above-mentioned nutrients, nitrogen (N) naturally has a large impact on potato yield. Though excessive N supply can reduce tuber maturity and quality, e.g. decreasing starch while increasing sucrose, free amino acids and reducing sugars, it is the nutrient with the greatest influence on tuber weight. N is primarily taken up during the tuber bulking phase, but it is also important for canopy development earlier in the growing season (Koch et al., 2019b).

In order to ensure optimal yield and quality of potato, it is therefore important to avoid nutrient deficiencies during the growing season. However, potatoes have a sparse and shallow root system, with up to 90% of roots located in the uppermost 25 cm of the soil (Tanner et al., 1982). This reduces their access to nutrients with limited mobility in soil, such as P, and results in a low P use efficiency. Placing P in or between the rows during planting increases root interception of the applied P, thus improving the P use efficiency (Hopkins et al., 2014). Before planting, it is common practice to perform a soil analysis to estimate the availability of plant nutrients, even though soil analyses repeatedly have shown a poor correlation between the extractable soil P and the plant available pool of P (Mundus et al., 2017). The availability of soil P furthermore depends on soil texture and environmental factors such as humidity, temperature and precipitation during the growth period (Rosen et al., 2014; Potarzycki and Grzebisz, 2019; Koch et al., 2019b). P deficiency therefore often occurs as a temporal disorder caused by climatic conditions. The crop might in such cases benefit from supplementation of P fertilizers applied to the foliage or incorporated into the soil (Rosen and Bierman, 2008; Rosen et al., 2014).

However, P deficiency often occurs without visible symptoms, and can be hard to diagnose by visual inspection (Carstensen et al., 2018a; Carstensen et al., 2018b). Monitoring the nutritional status and predicting yields can instead be done during the growing season by measuring nutrient concentrations in leaves or petioles. It has traditionally been reported that the youngest fully expanded leaf (YFEL) should have a P content above a critical P concentration of 0.22% at tuber bulking (Tindall et al., 1993; Rosen et al., 2014). The critical P concentration is the concentration below which the plant is considered P deficient, and the yields are expected to be affected. The YFEL is chosen due to its developmental stage, as it has a high photosynthetic activity but is not yet fully transformed from a net importer to a net exporter of photosynthates (Walworth and Muniz, 1993; Katoh et al., 2015; Grzebisz et al., 2018). Yet, regularly monitoring the nutrient composition can be both time consuming and expensive. Furthermore, it can be questioned how useful this classical approach is in potato, as recent research suggests it is insufficient to rely on macronutrient concentrations of the YFEL to predict yields (White et al., 2018; Grzebisz et al., 2020).

As an alternative to measuring nutrient concentrations, chlorophyll *a* fluorescence can diagnose physiological deficiencies of certain nutrients, when they affect nutrient specific functions of the photosynthetic machinery (Frydenvang et al., 2015; Carstensen et al., 2018a). Measurements of chlorophyll *a* fluorescence can be used to create a fluorescence transient, which is a polyphasic, time-dependent emission of fluorescent light, also known as an OJIP transient. In the OJIP transient, the fluorescence initially increases in three distinct steps (the O-, J-, and I-steps) before peaking (the P-step) at the maximum fluorescence (Fm) and subsequently decreasing. These steps are caused by discrete events in the linear electron transport from photosystem II (PSII) to photosystem I (PSI) and will be affected in different ways by distinct disruptions in the electron transport chain.

The P-predict parameter is a new chlorophyll *a* fluorescence parameter, which is derived from the fluorescence transients and has been developed to characterize P status in barley grown under different levels of P deficiency (Frydenvang et al., 2015; Carstensen et al., 2018a; Carstensen et al., 2018b). P deficiency is correlated with specific changes in the fluorescence transient and appears to be independent on most other nutrient deficiencies. Under P deficiency, a rapid lumen acidification decreases the rate of plastoquinone oxidation and erases the I-step (Carstensen et al., 2018a), resulting in a smooth curve between the J- and P-steps. The P-predict value is calculated largely based on the degree of I-step deletion (Frydenvang et al., 2015).

Chlorophyll *a* fluorescence can also be used to detect Manganese (Mn) deficiency, because it decreases the quantum yield (Fv/Fm) of PSII and results in a low maximum fluorescence. As a result, Mn deficiency distorts the shape of the fluorescence transients (Schmidt et al., 2016b). The effects of P deficiency on a fluorescence transient will therefore be obscured if it occurs simultaneously with Mn deficiency, preventing detection of both deficiencies at once.

Environmental stressors, such as excess light, extreme temperature or drought, can result in photoinhibition which affects chlorophyll *a* fluorescence patterns and the resulting P-predict values (Ruban, 2016; Guidi et al., 2019). The P-predict approach has primarily been developed and used in controlled conditions in greenhouses and climate chambers. However, field grown crops are inevitably exposed to various types of stresses during a growing season. Testing the applicability of the P-predict method in real field settings across different crop species and climates is therefore required to ensure the method’s suitability for standard agronomical practices. It should be underlined that this method is suitable for diagnosing the immediate plant P status, but is unable to provide a prognosis of how the future plant P status might develop.

This study aims to investigate the P-predict responses under field conditions, and to determine best practice for diagnosing P deficiency in a P sensitive crop such as potato. This was done by following the nutrient concentrations at different leaf positions throughout the growing season and correlating the P concentrations with diagnosis of the P status based on chlorophyll *a* fluorescence analyses.

## 2 Materials and methods

### 2.1 Field experiment

The experiment was conducted in western Jutland, Denmark (55°36’13.4”N, 8°57’09.0”E) on a field where P deficiency had previously been observed in potatoes. Soil samples from the top 25 cm were randomly collected, air dried and sieved through a 2 mm mesh. The soil samples were analyzed by standard procedures and included: texture, pH, Olsen-P and ammonium extractable Mg and K at a commercial laboratory (Agrolab, Germany) (**Table 1**). Furthermore, P availability was determined by the C_DGT_ technique as presented by Tandy et al. (2011) and Christel et al. (2016) (**Table 1**). Seed potatoes *Solanum tuberosum* L., cv. Kuras were planted with a row distance of 0.75 m and a distance between tubers of 0.2 m. The experimental site was irrigated when there was no precipitation. Fertilizer was placed between the rows (10 cm from the tubers and 5 cm below tuber level) while planting. The following fertilization rate was used: 136.5 kg N ha^-1^, 250 kg K ha^-1^, 60 kg Mg ha^-1^, 170 kg S ha^-1^ and it was provided as diammonium phosphate, N-S (27-4) and patentkali (25% K, 6% Mg, 17.5% S). Two soil P fertilizer treatments were used: P+ and P-. P+ was supplied with 30 kg P ha^-1^, while P- did not receive P fertilizer.

**Table 1:** Soil properties at the field site in Hovborg, Denmark.

Soil content	Value
Clay <0.002mm (%)	4.3
Silt 0.002-0.02mm (%)	0.3
Fine sand 0.02-0.2mm (%)	22.9
Coarse sand 0.2-2mm (%)	69.4
SOM (Soil Organic Matter) (%)	3.1
pH (0.01 M CaCl_2_)	5.4 ± 0.0
Olsen-P (mg P kg^-1^ soil)	4.6 ± 0.4 (>2.0)
C_DGT_ (µg P L^-1^)	66.8 ± 2.2 (>65)
K (mg K kg^-1^ soil)	29.5 ± 0.9 (>40)
Mg (mg Mg kg^-1^ soil)	35.5 ± 0.6 (>40)

Values in parenthesis indicate standard thresholds for sufficient soil nutrient concentrations. Results presented as mean ± SEM (n = 4).

Soil moisture (0-20 cm below the soil surface) and soil temperatures (6 cm below the surface, at the soil surface and 15 cm above ground) were monitored using two Standard TMS dataloggers (Tomst, Czech Republic) throughout the entire growing season. On the days when tissue sampling and chlorophyll *a* fluorescence measurements were made, the photosynthetically active radiation (PAR) was recorded for a period of at least 3 hours using a universal light meter (ULM-500, Heinz Walz GmbH, Germany).

### 2.2 Elemental analysis of potato leaves

All leaf positions were harvested from 4 field grown potato plants at each sampling date, giving a total of 4 independent replicates of each leaf position. Sampling occurred twice in June at 51 and 58 days after planting (DAP) during the tuber initiation stage, and twice in August at 99 and 113 DAP, during the tuber bulking stage. The leaves were dried at 50°C on the day of harvesting. Ultra-wave digestion of plant tissue was performed in a mixture of 500 µL ultra-pure acid (70% HNO_3_) and 250 µL 15% H_2_O_2_ under an inert gas (N_2_) at a pressure of 45 bars and 240°C for 15 min (Ultrawave, Milestone Inc., USA). When leaf tissue dry weight exceeded 150 mg, the leaves were pulverized and homogenized before taking a sub-sample. All samples were analyzed on a 5100 DW ICP-OES (Agilent Technologies, USA) with multi-element detection. Data were validated with the analysis of seven replicates of certified reference material (Apple leaves, NIST 1515, National Institute of Standards and Technology, USA). Only elemental data with accuracies and precision better than 95% were included (Chen et al., 2020).

### 2.3 Chlorophyll *a* fluorescence analysis

Chlorophyll *a* fluorescence analysis was performed on all leaf positions (n = 4) before harvesting at 51, 58, 99 and 113 DAP, using a HandyPEA chlorophyll fluorometer (Hansatech instruments, UK). Prior to measuring, leaves were dark adapted for 25 minutes using Hansatech leaf clips (Hansatech instruments, UK). Leaves were exposed to continuous saturating actinic light (3000 µmol m^-2^ s^-1^) for 10 seconds. The resulting fluorescence emission was recorded by a PIN photodiode to construct a fluorescence transient (Kautsky and Hirsch, 1931). The transients were created by double normalizing the data between F0 and Fm to get the relative fluorescence, V(t), using the formula:


$$V(t)=\frac{F(t)-F0}{Fm-F0}$$

F0 and Fm correspond to the minimal fluorescence at time 0 and the maximum fluorescence, respectively, and F(t) is the measured fluorescence at time *t*. Furthermore, the fluorescence transients were used to evaluate the plant P status, by preprocessing and analysis using a Python 3.9 script based on a partial least square (PLS) model to quantify the level of P deficiency (Frydenvang et al., 2015) (Patent EP3049792B1). P-predict values can provide an indication of plant P status and identify P deficiency. Plants are considered P deficient at P-predict<0.40 and P sufficient at P-predict >0.65 while P-predict values in the range 0.40-0.65 indicate intermediate P status (Frydenvang et al., 2015; Carstensen et al., 2018a).

## 3 Results

### 3.1 Variability of leaf nutrient content

**Figure 1:**
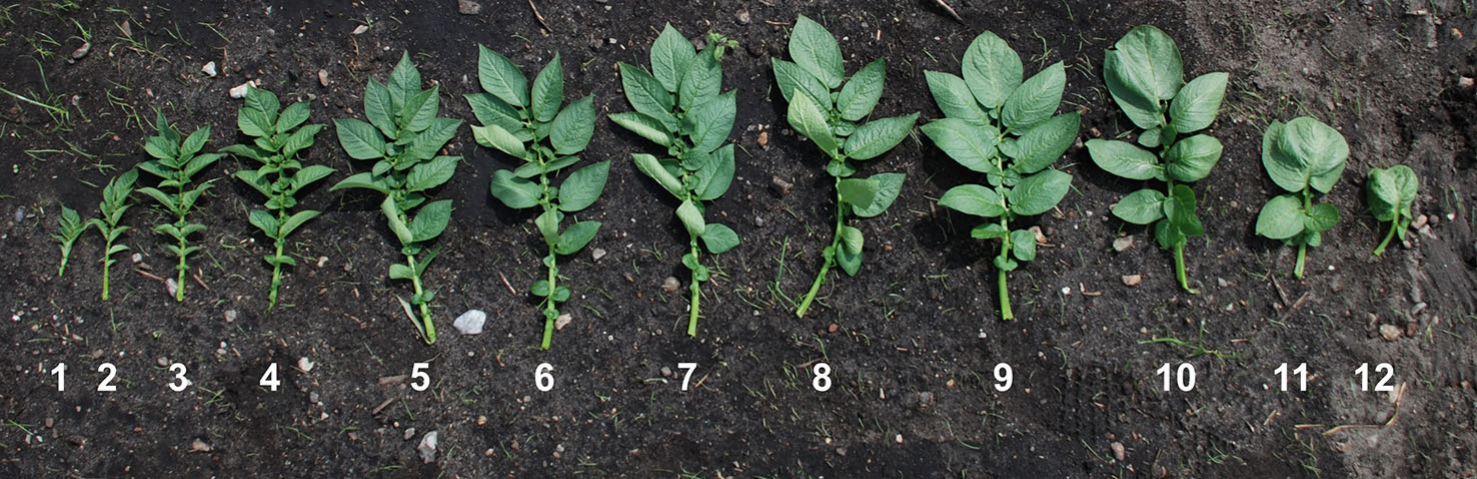
Leaf positions. One replicate of all leaves from a single plant at 51 days after planting. Numbers indicate leaf position, 1 being the youngest and 12 the oldest.

The experimental field site was chosen due to a historical yield response to P fertilization, which was confirmed by a pot trial that showed an increased barley biomass following P fertilization (data not shown). The soil has a high percentage of coarse sand (**Table 1**), which is suitable for potato cultivation. The soil type has a low water holding capacity often resulting in low P availability and drought stress in absence of irrigation, and for this reason irrigation was implemented. The volumetric soil moisture was maintained above 25% for the duration of the experiment (**Supplementary Figure S1**), thus exceeding the field capacity for the sandy soil (Zotarelli et al., 2019). Soil tests for P status showed that the soil was near deficiency according to C_DGT_, which is the most reliable indicator of plant available soil P (Mundus et al., 2017), while it had sufficient P levels according to the Olsen-P value (Rubæk, 2015) (**Table 1**). The field experiment was fertilized according to standard practices and recommendations based on the soil tests, and the plants presented no visible symptoms of any nutrient deficiencies (**Figure 1**).

**Figure 2:**
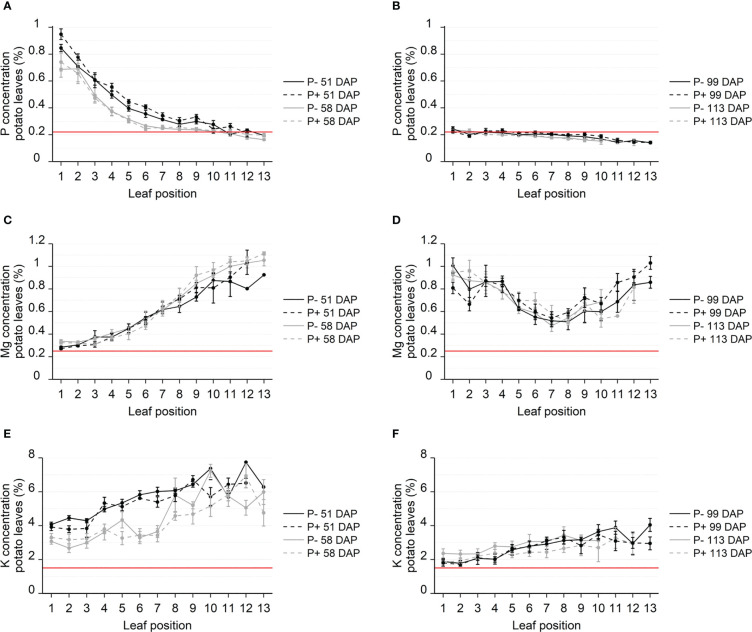
Concentration of P **(A, B)**, Mg **(C, D)** and K **(E, F)** in leaves from field grown potato. Leaves sampled from four replicate plants throughout the growing season, 51-113 days after planting (DAP), during tuber initiation **(A, C, E)** or tuber bulking **(B, D, F)**. Nutrient concentrations were analyzed using ICP-MS and reported in %. Leaf positions specify the location of the leaf on the plant with lower numbers being the topmost, younger leaves and higher numbers indicating increasing leaf age. Red lines show standard threshold values for deficiencies for each nutrient in potato. Error bars show SEM (n = 4).

The elemental composition was measured in all leaves from four randomly selected plants at each sampling date and showed striking patterns of nutrient concentrations depending on leaf position and sampling date (**Figure 2**). P fertilization had a significant effect on the P concentration at the individual leaf positions during tuber initiation on the first sampling date 51 DAP (P<0.001), but no significant differences were found 7 days later, nor at the tuber bulking stage 99 and 113 DAP. During tuber initiation, halfway through the growing period at 51 DAP, P concentrations were very high in the youngest leaves (0.85-0.94% in leaf 1) and decreased markedly with increasing leaf age. Leaf 5 had P concentrations of 0.40-0.44%, while leaf 11 had P concentrations of 0.20-0.26%, which is within 1 standard deviation of the reported critical P concentration of 0.22% (**Figure 2A**). One week later, at 58 DAP, P concentrations in leaves 3-7 had decreased by a remarkable 19-40% compared to the previous week, with concentrations in older leaves (leaf 7-13) near the critical P concentration. During tuber bulking in the later part of the growing season (99 and 113 DAP), P concentrations in all leaves had stabilized at or below the critical P concentration (**Figure 2B**). Furthermore, no differences were found in tuber P concentration between P fertilization treatments (**Supplementary Table S1**).

The distribution patterns observed for Mg and K concentrations were opposite to the ones observed for P during tuber initiation (**Figure 2**). Mg and K did, however, resemble those of phloem immobile nutrients such as Ca, Mn and B, while the nutrients S, Fe, Zn and Cu all differed in distribution patterns with seemingly no relation to their phloem mobility (**Supplementary Figures S2** and **S3**). While the younger leaves had the highest P concentrations, they had the lowest Mg and K concentrations, (**Figures 2C, E**). The youngest leaves had Mg concentrations slightly above the critical Mg concentration of 0.25%, while Mg concentrations in leaf 11-13 were four times higher, peaking at around 1%. Similar to Ca and Mn, Mg concentrations did not decrease between 51 and 58 DAP. During tuber bulking, Mg concentrations in leaf 7 had fallen to 0.51%, while both older and younger leaves contained high levels of Mg (up to 0.81-1.0%) (**Figure 2D**). The K concentration decreased systematically over time. In the span of one week during tuber initiation, K concentrations were significantly reduced in all leaves (**Figure 2E**), similar to the P concentrations. The K concentrations had fallen by 50% at the time of tuber bulking 113 DAP (**Figure 2F**). In the time between tuber initiation and tuber bulking, the K concentration decreased from 4% to 1.7% in the youngest leaves and from 7% to 4% in the oldest.

These data indicate that the results of leaf nutrient analyses are highly dependent on leaf position and time of sampling, more so than on the availability of nutrients in the soil.

### 3.2 Diagnosis of P status with chlorophyll *a* fluorescence

**Figure 3:**
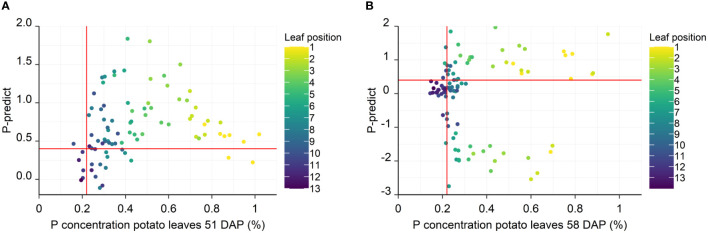
P-predict values correlated with the leaf P concentration at 51 DAP **(A)** and 58 DAP **(B)**. Each point corresponds to one measured leaf, color coded for leaf position. The vertical red line indicates the P concentration threshold for P deficiency at 0.22%, while the horizontal line represents the P-predict level indicating P deficiency at 0.4. Points located in the upper-left and lower-right are thus misclassified as being P sufficient or P deficient, respectively. 51 DAP: n = 90, 58 DAP: n = 97.

Prior to the elemental analysis shown in **Figure 2**, chlorophyll *a* fluorescence was measured to generate P-predicts for all leaf positions. P-predict values are used to estimate the P status in plants, where values below 0.4 indicate a P concentration below the critical P concentration. Correlating P-predicts with leaf P concentrations during tuber initiation (51 DAP) shows that according to both P concentrations and P-predict values, 72% of the leaves were not P deficient (**Figures 2A**, **3A**). Only 8% of the leaves were classified as being P deficient according to both P-predict and leaf concentration, while 19.5% of the leaves were misclassified by P-predicts. Strikingly, the rate of P-predict misclassification increased to 43% at 58 DAP. Most of the misclassifications on both days came from P-predicts indicating P deficiency, although leaf concentrations were above the critical P concentration. At 51 DAP, most of the leaves misclassified as deficient by P-predict had relatively low P concentrations between 0.22% and 0.4%. At 58 DAP, leaves 1-11 were frequently misclassified as P deficient by P-predict, irrespective of their P concentration (**Figure 3B**). At 51 DAP, the P-predicts were highest in the YFELs at position 4-5, while the youngest, not fully expanded, leaf positions 1-3 showed decreasing P-predict values despite having the highest P concentrations. Two replicates of leaf 1 had P concentrations of nearly 1% but were classified as deficient by their P-predicts.

Given the increasing number of misclassifications, it is clear that the reliability of the P-predict as a diagnostic tool was affected prior to day 58. This indicates a disturbance in the photosynthetic machinery, as chlorophyll *a* fluorescence is tightly correlated with the linear electron flow from PSII to PSI. Besides nutrient deficiencies, fluorescence emissions can also be affected by stressors such as light or temperature. The irradiance was significantly higher on day 58 compared to day 51, which was likely contributing to the higher rate of misclassified P-predicts (**Supplementary Figure S4**). During fluorescence measurements on day 51, the irradiance fluctuated around 600 PAR, with single spikes at 1600, while the irradiance was significantly higher at 1400-1500 PAR on day 58, with short dips when clouds passed. Similarly, the air temperature was 26°C on day 50 and 51, but 32°C on day 57 and 58 (**Supplementary Figure S5**).

**Figure 4:**
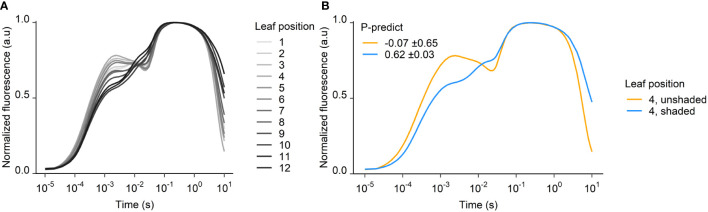
Fluorescence transients from 58 DAP at **(A)** different leaf positions and **(B)** shaded and unshaded YFEL. P-predict ± SEM is inserted for shaded and unshaded leaves in **(B)** (n = 4).

The fluorescence transients serving as input to the P-predict calculations were severely distorted on day 58, especially in the young and mid-level leaves (positions 1-7). These leaves represent the most sun-exposed parts of the plant, and their fluorescence transients lost their J- and I-steps and instead developed D-dips where the I-step is usually located. The transients of the older leaves, deep in the shaded canopy looked normal (**Figure 4A**).

To avoid light stress and potential photoinhibition, some plants were shaded from day 51, which resulted in normal transients and reliable P-predict values on day 58. The transients of shaded leaves showed distinct OJIP steps on day 58 and were markedly different from the distorted transients in the same leaf positions in unshaded plants (**Figure 4B**). The shading decreased the irradiance from 1400 to 600 PAR, while the temperature inside and outside the shaded areas were nearly identical. P-predict values ranged from –2 to 2 (average –0.07 ± 0.65) in the unshaded leaf 4, while shading of leaf 4 improved P-predict values to 0.62 ± 0.03. Shading thus enabled a correct P-predict diagnosis (**Figure 4B**).

## 4 Discussion

### 4.1 Distinct patterns of remobilization

In this study, we observed a steep gradient of leaf P concentrations through the canopy. The youngest leaves had a high P concentration during tuber initiation, while the older leaves had concentrations around or just above 0.22% (**Figure 2A**). When the nutrient supply becomes limiting, it is often observed that young leaves retain high nutrient levels at the expense of older leaves due to remobilization (Smith and Loneragan, 1997). In the current experiment, the observed P depletion of the older leaves was caused by a substantial remobilization of P from source to sink tissue, i.e. to the young leaves and the developing tubers of the potato plant. During tuber initiation, the P concentration in the YFEL fell by 25-33% in the span of just one week, indicating a large remobilization from shoot to tuber. Later in the growing season, during the tuber bulking period, all leaves ended up near or just below the critical P concentration of 0.22% which indicated that the shoot P was remobilized to the tubers and settling at the critical P concentration. The timing and leaf sampling prior to measurements is therefore incredibly important for a meaningful interpretation of leaf analysis data.

Interestingly, K and Mg distribution presented opposite patterns compared to P. The concentration of K and Mg were highest in the older leaves and did not exhibit deficiency levels in the late season. This indicates a sufficient K and Mg availability, even though the concentration of K fell significantly between 51 and 58 DAP and was close to the critical K concentration in the youngest leaves 113 DAP.

It is remarkable that the distribution patterns of K and Mg resemble those of the phloem immobile nutrients Ca and Mn during tuber bulking, despite Mg and K presumably having a high phloem mobility similar to that of P (**Supplementary Figures S2**, **S3**). P concentration in tubers at harvest was 0.15% (**Supplementary Table S1**), and from **Figure 2B** it is clear that P was remobilized fully until it reached the critical P concentration of the shoot. This did not occur to the same extent for Mg and K and indicates that the tubers were a stronger sink for P than for Mg and K (**Figures 2D, F**). This is likely because the primary involvement of K and Mg in starch synthesis occurs through phloem loading of photosynthates in the shoots. P is also engaged in phloem loading but is remobilized to the tubers due to a key-functionality in activating enzymes of the starch synthesis pathway in the amyloplasts (MacNeill et al., 2017).

However, although we observed remobilization of P from old to younger tissue, it is not clear whether P availability was actually limited in this experiment. In fact, no differences could be observed between fertilized and unfertilized plots, neither in P-predicts, P concentrations nor in the final yields. Across both treatments, we only observed P concentrations below 0.22% in the older leaves on the later sampling dates. The missing response to P fertilization could indicate that the availability of P from residual pools in the soil made the applied fertilizer P redundant. This has previously been observed during optimal water conditions, and is therefore a likely observation in the current study (Potarzycki and Grzebisz, 2019). It is also possible that the buffering capacity of the soil was sufficient to supply the plants with enough P to satisfy the functional requirement, although it would be unexpected, as the field was chosen due to a history of P fertilization responses.

The lack of fertilizer effect could also imply that the applied P was immobilized and not available for uptake by the roots. It is therefore unclear if the low P concentrations measured in the older leaves in the early growing season, and in all leaves in the late growing season, should be classified as a nutrient deficiency or a normal physiological consequence of massive P remobilization to the tubers. Once the shoot has become a P source for tuber development, any further P uptake is not likely to be reflected in leaf concentrations as it will be directed towards the tubers. The P response, which was not achieved under field conditions, could certainly have been obtained under controlled conditions (climate chamber or greenhouse). However, this would clearly have omitted all interactions with the environment that are so essential for an appropriate and “real-life” assessment of the P-predict method.

### 4.2 Diagnosing nutrient deficiencies

The nutrient concentrations are highly dynamic and observed to change drastically within a few days, which essentially prevents meaningful data interpretation based on single leaf analysis. Furthermore, the nutrient demand of plants fluctuates during a growth cycle, and a tissue analysis cannot foresee if deficiencies will arise later in the season. The tissue concentration would therefore need to be regularly monitored, which is both labor and cost intensive. The critical P concentration of 0.22% in the YFEL is widely used as a threshold for diagnosing P deficiency (Rosen et al., 2014). This critical P concentration is set for mid- to late season potatoes, but the critical concentration is higher in the early growth stages (Walworth and Muniz, 1993; Huett et al., 1997). The critical concentration of 0.22% corresponds well with the concentration at which most leaves are classified as deficient by P-predict. The P concentration in the YFEL of P sufficient potato plants decreases from 0.6% in early season to 0.4% in mid-season and 0.2% in late season (Walworth and Muniz, 1993). A concentration of 0.3% might therefore be indicative of P deficiency in the early growth phases, but not in the late growing season. In this study, the YFEL (leaf 4) had a P concentration around 0.4% in the mid-season (51 and 58 DAP), which would also be deemed sufficient by these standards.

When chlorophyll *a* fluorescence is measured, thresholds are also used to evaluate the P status of plants. The fluorescence parameter P-predict is used to indicate when P deficiency physiologically affects the functionality of the photosynthetic processes in the plants (Frydenvang et al., 2015; Carstensen et al., 2018a). In this study, the different leaf positions presented a wide range of P status, from severely deficient in the older leaves late in the season to very high concentrations in the youngest leaves early in the season. A positive correlation between leaf P concentration and P-predict was observed for leaf positions 4-13 (**Figure 3A**). However, the three youngest leaves had lower P-predicts despite having higher P-concentrations. These leaves are not yet fully evolved and have thus not yet transitioned from being sink to source tissue which is reflected in the fluorescence signal. Measuring a fully expanded leaf is therefore necessary for correct classification of P status.

The advantages of chlorophyll-based diagnosis in field settings, as an alternative to measuring the nutrient concentration in leaves, include the cheap cost and rapid on-site classification compared to plant tissue analysis by external laboratories, where it can take up to several weeks to receive the result of tissue sampling. Obtaining data about the crop nutrient status in real time will allow immediate fertilization efforts to alleviate P deficiencies, before the deficiency can affect yield outcomes.

It can be valuable to measure tissue P concentration during the growing season to add P fertilizers in response to low P status (Rosen et al., 2014), and P concentration and yields have been found to correlate (Fernandes et al., 2014). Yet, research has also shown that macronutrient concentrations in leaves can be poor indicators of yield, for both P (White et al., 2018) and N (Grzebisz et al., 2020). White et al. (2018) grew 66 potato genotypes and found a positive effect of P fertilization on both yield and leaf P concentration. However, within the tested cultivars there was no correlation between P concentration in the YFEL and tuber yields. In previous studies, P concentration and P-predict could only predict yield effects in the first few weeks of the barley growing season (Carstensen et al., 2018b). Later in the season, the size of the plant had adjusted to the available P levels, and the P status of each leaf was therefore at sufficient levels, despite a significant yield response. In this study, a transient P effect of P fertilization was observed during the early tuber initiation phase (**Figure 2A**) but disappeared at the next sampling time 7 days later. The transient P response indicates that the plant adapts its size to the available P. However, in this case P fertilization would be expected to influence measured yield which was not found.

### 4.3 Photoinhibition and shading

Despite the high correlation between leaf P concentration and P-predict on day 51, field grown crops are often exposed to environmental stresses which may interfere with chlorophyll *a* fluorescence. In this study we observed that high solar irradiance (>1400 PAR) on measurement days impaired the reliability of the fluorescence-based diagnosis of P status. The chlorophyll *a* fluorescence response in the leaves were severely affected, leading to erroneous P-predict estimates of P status. The fluorescence transients showed indications of photoinhibition, including the development of D-dips in the OJIP transients following the I-step at approximately 0.07 seconds (**Figure 4**). The D-dips that developed at day 58 under high irradiance might be related to degradation of the Mn cluster of the oxygen evolving complex at PSII (Pospíšil and Dau, 2000; Schmidt et al., 2016a). D-dips have also been observed in transients of Mn deficient plants, which are severely affected by photoinhibition (Schmidt et al., 2016b). Photoinhibition is a light-induced inhibition of PSII. It is a mechanism plants employ to protect the photosystems from photodamage caused by excess energy from absorbed light and is part of non-photochemical fluorescence quenching (NPQ). The primary components of NPQ respond to thylakoid lumen acidification and reverse quickly during dark adaptation. Photoinhibition (qI), however, is only induced over prolonged exposure to excess light, and is caused by inactivation or degradation of the D1 subunit of PSII, or from permanent damage to the photosynthetic systems (Ruban, 2016; Guidi et al., 2019). It therefore reverses slowly, over the span of hours or days, as the reversion depends on D1 repair mechanisms. In order to understand the cause of the distorted transients at the mechanistic level, detailed PAM fluorometry would be required to identify the individual NPQ components (qE, qT, qI). Moreover, to further elucidate the correlations between high light intensity and the shape of the distorted transients, it would be necessary to perform experiments with a range of light levels and plant P tissue levels under controlled growth conditions. Such experiments could determine the intensity at which high light causes distortion at a given P status, and how low the intensity should be to subsequently restore the fluorescence transients for diagnosis of nutrient status.

To understand the cause of the distorted transients in the field, some plants were shaded for one week prior to measuring chlorophyll a fluorescence on a day with high light intensity. In this experiment it was observed that shading the plants resulted in fluorescence transients comparable to those obtained at low light, either measured on a cloudy day or on the leaves shaded in the canopy (**Figures 3**, **4**). Leaves deep in the canopy will always be shaded, and as a result their transients will be unaffected by photoinhibition, which consequently makes them useful for diagnosing P status. However, these older leaves without exposure to high light were P depleted early in the season due to P remobilization, and a diagnosis based on these leaves would therefore indicate P deficiency, even when the plant might be fully supplied with P. In effect, measuring chlorophyll a fluorescence for diagnosis of nutrient status should be avoided on days with extreme light intensity. If this is not possible, it will therefore be necessary to shade the plants for several hours prior to chlorophyll *a* based diagnosis of P status.

In order to use these techniques for reliable diagnosis of P deficiency and fertilizer planning, P status needs to be monitored throughout the season. Due to the highly dynamic P concentrations, meaningful interpretation is incredibly challenging when using the traditionally established critical P concentrations. Chlorophyll *a* fluorescence analysis provides a cheaper and immediate diagnosis, but threshold values for P-predict at the different growth stages are not yet established. Furthermore, interpretation of both methods is complicated by the highly dynamic P concentrations and thresholds for P deficiency which vary throughout the growth season.

## 5 In conclusion

While the traditional practice of measuring tissue concentrations gives an exact measure of the P content of the plant, the interpretation is not straightforward. The nutrient concentrations in potato plants are highly dynamic, with large variations over time and across different leaf positions. Attention to sampling time is therefore incredibly important for a meaningful interpretation, as P concentrations fell by 19-40% in the span of just 7 days during tuber initiation. The picture is further complicated when attempting to characterize the status of several nutrients at once, as the distribution of different nutrients are distinctly different, even when comparing nutrients which are generally considered to be equally phloem mobile, such as P, Mg and K. In the current experiment, the tubers acted as a strong sink for P and to a lesser degree K, consequently depleting the shoot of these nutrients to deficiency levels at the end of the growing season. Thus, frequent sampling and analysis are required to monitor the P status of potatoes and to prevent P deficiency from occurring.

This study shows that chlorophyll *a* fluorescence is able to rapidly estimate the bioactive P pool across a range of leaf positions to characterize the P status of field grown potatoes. However, in the field high solar irradiation may affect the photosynthetic machinery, induce photoinhibition, and thereby disturb the resulting fluorescence signals. It was shown that photoinhibition may be reversed by shading the plants. Future research should investigate the interactions between NPQ and leaf P status at different PAR to potentially develop an algorithm capable of delivering P-predicts at high light.

## Data availability statement

The raw data supporting the conclusions of this article will be made available by the authors, without undue reservation.

## Author contributions

ST and AS designed and carried out the experiments. AS did the data analysis, ST and PM wrote the manuscript, and SH designed the experiments and reviewed the manuscript. All authors contributed to the article and approved the submitted version.
