# Supplementary material for: Leaf age and light stress affect the ability to diagnose P status in field grown potatoes
Source: Front Plant Sci. 2023 Apr 20;14:1100318. doi: 10.3389/fpls.2023.1100318 (PMC10157199; doi:10.3389/fpls.2023.1100318)
Supplement: Supplementary file 1 [file DataSheet_1.docx]

Supplementary Material

# Supplementary Figures and Tables

Supplementary Table 1. Nutrient concentrations in yield of potato at 175 DAP (n=8). Yield pr plant is given for plots with (P+) or without (P-) P fertilization.

|  | Nutrient concentrations (μg/g) | | Yield pr plant   (Kg) | |
| --- | --- | --- | --- | --- |
| P+ | 1,541.95 | ± 104.67 | 1.95 | ±0.07 |
| P- | 1,529.51 | ± 70.84 | 1.89 | ±0.04 |
| K | 14,925.92 | ± 482.25 |  |  |
| S | 1,047.41 | ± 46.63 |  |  |
| Mg | 787.59 | ± 25.83 |  |  |
| Ca | 247.11 | ± 8.12 |  |  |
| Fe | 10.56 | ± 0.52 |  |  |
| B | 4.52 | ± 0.12 |  |  |
| Mn | 3.10 | ± 0.14 |  |  |
| Zn | 11.22 | ± 0.34 |  |  |
| Cu | 2.18 | ± 0.15 |  |  |


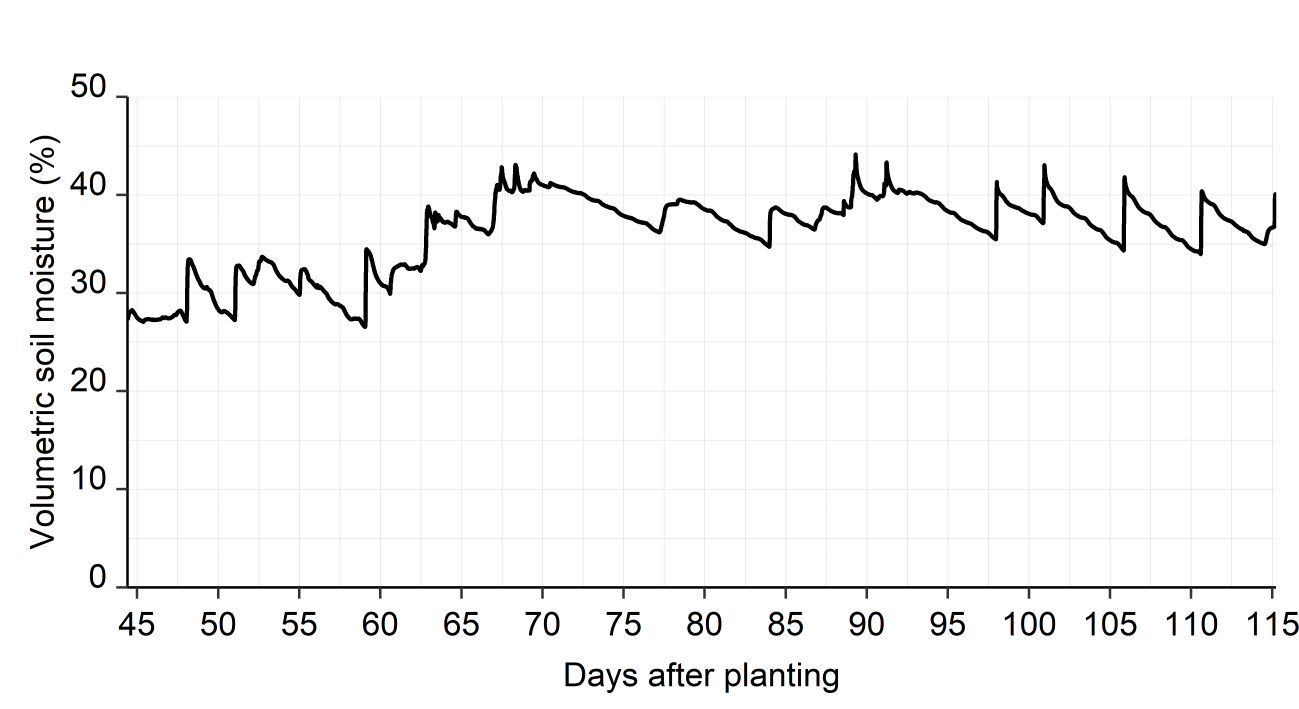


**Supplementary Figure 1.** Volumetric soil moisture (%) between day 45 and 115 days after planting, measured 0-15 cm below soil surface in the row. Labels indicate when the day is starting (midnight) and is thus ending at midnight following day.


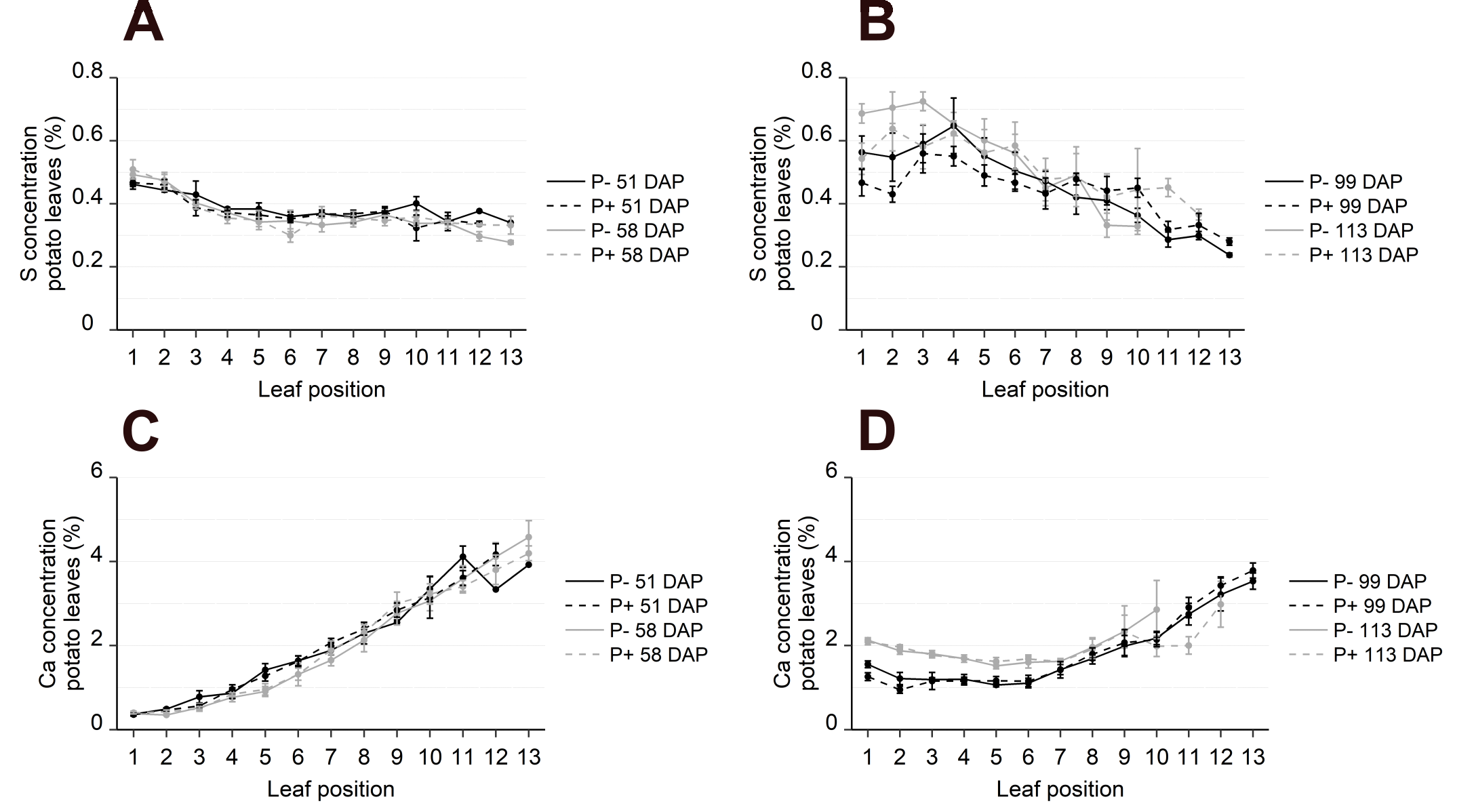


**Supplementary Figure 2.** Concentrations of S (**A-B**) and Ca (**C-D)** in leaves from field grown potato. Leaves sampled from four replicate plants throughout the growing season, at 51-113 days after planting (DAP), during tuber initiation (**A,C**) or tuber bulking (**B,D**). P+, but not P-, received P fertilization prior to planting. Nutrient concentrations were analyzed using ICP-MS and reported in %. Leaf positions specify the location of the leaf on the plant with lower numbers being the topmost, younger leaves and higher numbers indicating increasing leaf age. Error bars show SEM, n=4.


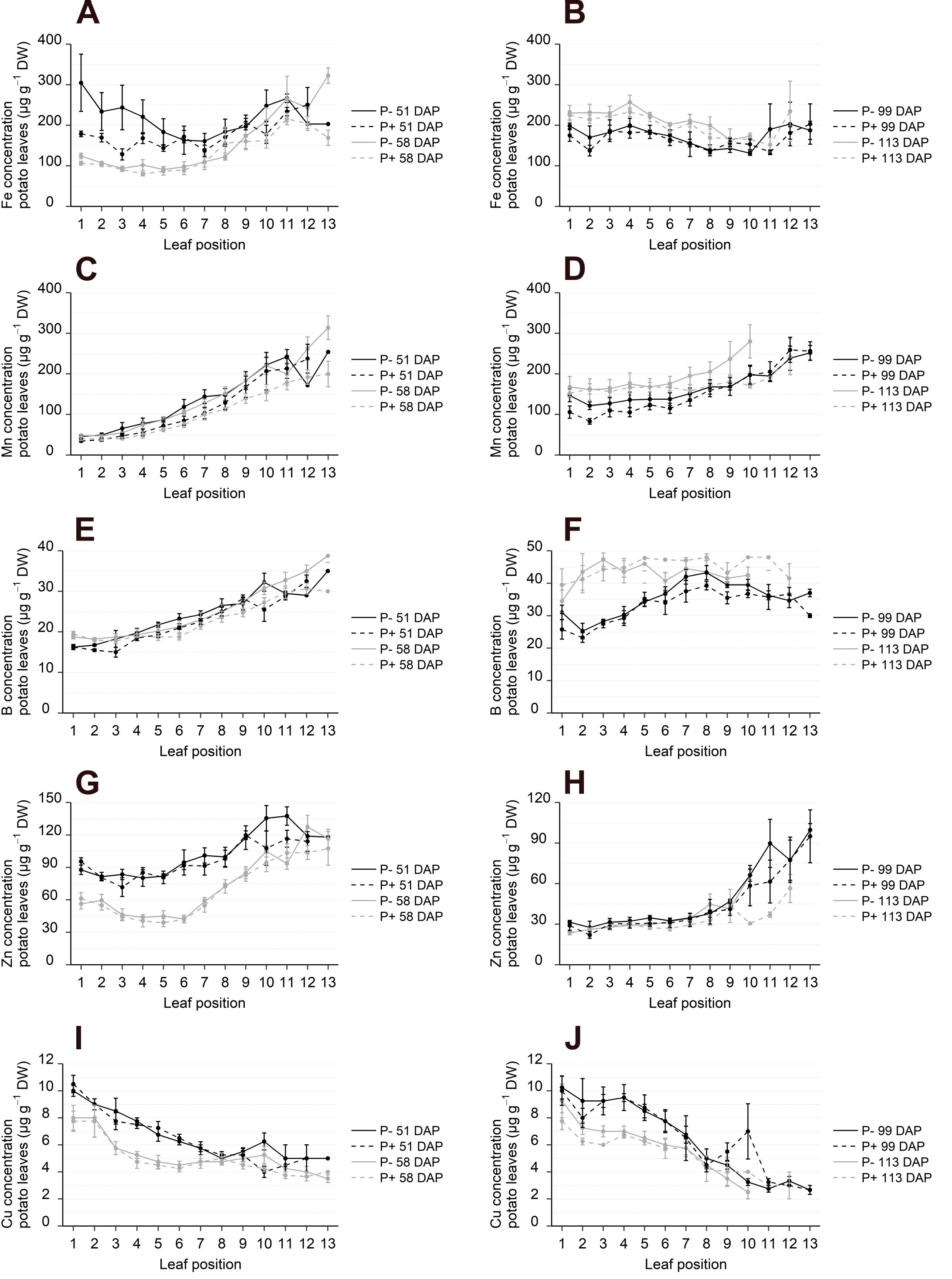


**Supplementary Figure 3.** Concentrations of Fe **(A-B),** Mn **(C-D),** B **(E-F),** Zn (**G-H)** and Cu (**I-J)** in leaves from field grown potato. Leaves sampled from four replicate plants throughout the growing season, at 51-113 days after planting (DAP), during tuber initiation (**A,C,E,G,I**) or tuber bulking (**B,D,F,H,J**). P+, but not P-, received P fertilization prior to planting. Nutrient concentrations were analyzed using ICP-MS and reported in μg g^-1^ dry weight. Leaf positions specify the location of the leaf on the plant with lower numbers being the topmost, youngest leaves and higher numbers indicating increasing leaf age. Error bars show SEM, n=4.


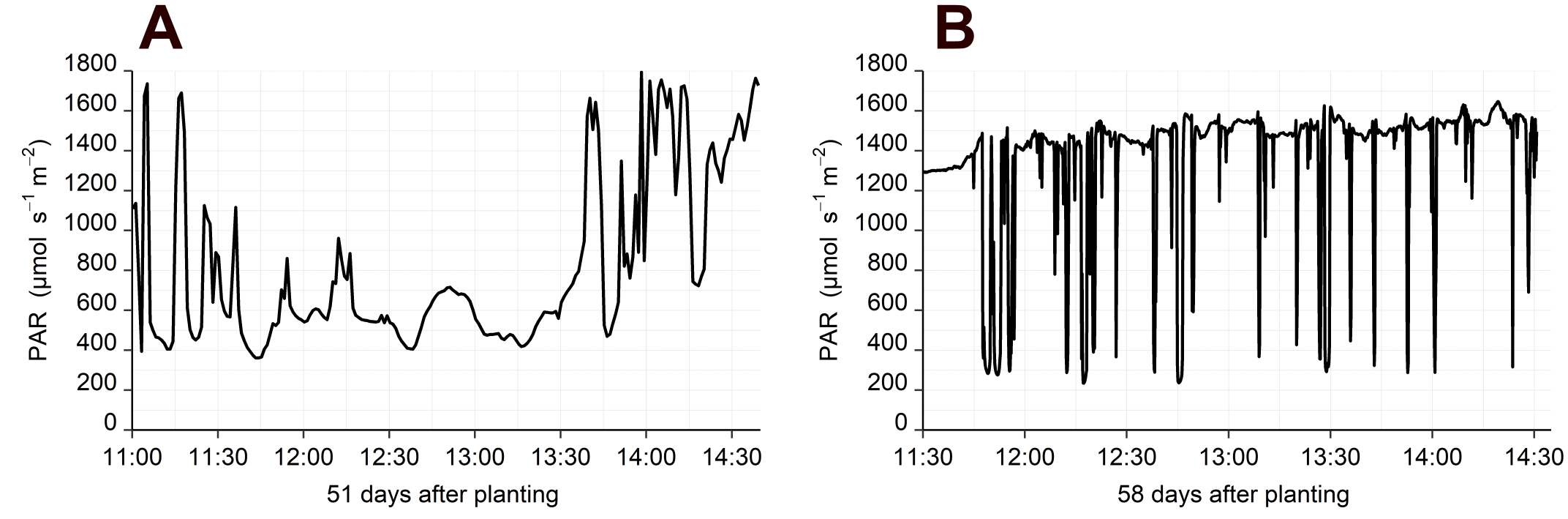


**Supplementary Figure 4.** Light intensity during the three measuring hours on 51 DAP (**A**) and 58 DAP (**B**).


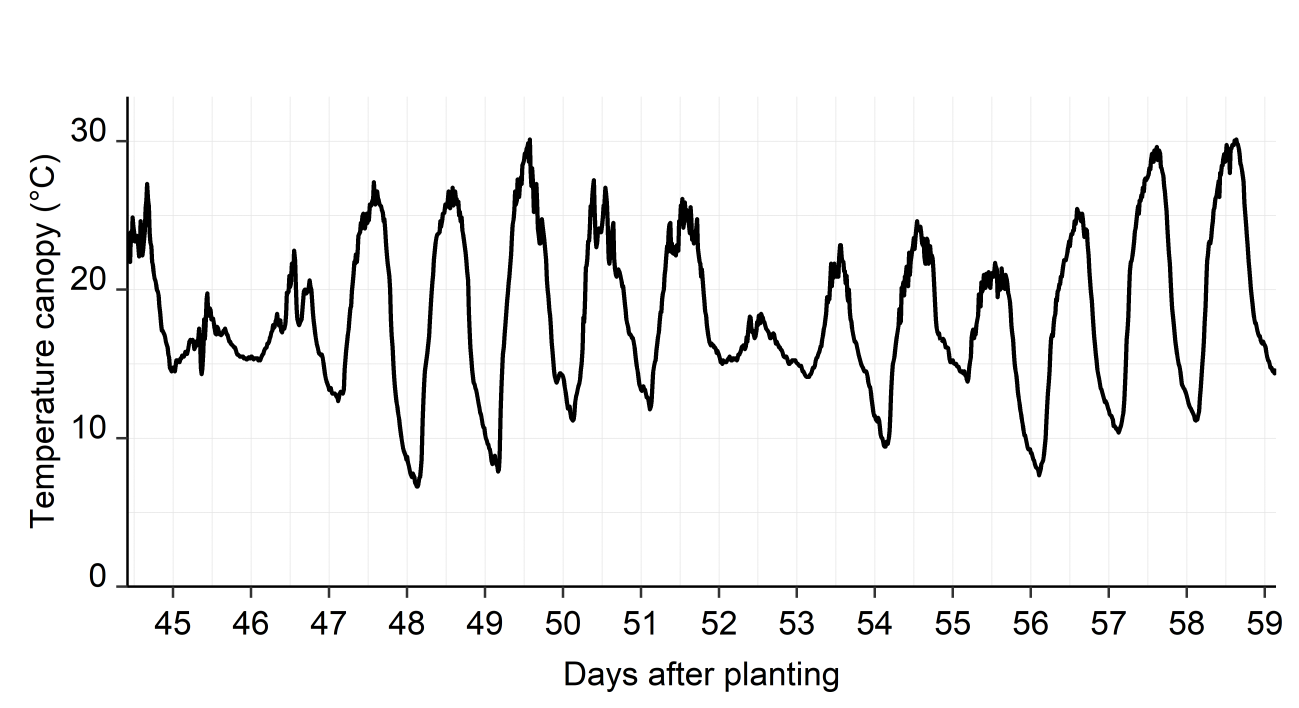


**Supplementary Figure 5.** Temperature shown between 44 and 58 days after planting, measured 15 cm above the soil surface in canopy. Labels indicate when the day is starting (midnight) and is thus ending at midnight following day 58.
